# CircRNA 0009043 suppresses non-small-cell lung cancer development via targeting the miR-148a-3p/DNAJB4 axis

**DOI:** 10.1186/s40364-022-00407-y

**Published:** 2022-08-16

**Authors:** Kelin She, Shaoqi Yu, Shushuai He, Wen Wang, Biao Chen

**Affiliations:** 1 Department of Thoracic Surgery, Hunan Provincial People’s Hospital, The First Affiliated Hospital of Hunan Nomal University, Changsha, Hunan 410005 China; 2 Cancer Research Institute, Central South University, Changsha, 410078 Hunan China; 3 Department of Thoracic Surgery, The Central Hospital of Shaoyang Affiliated to University of South China, 422000, Shaoyang, China

**Keywords:** Circular RNA, circ_0009043, NSCLC, microRNA, DNAJB4

## Abstract

**Background:**

Circular RNAs (circRNAs) are important regulators of the development and progression of non-small-cell lung cancer (NSCLC) and many other malignancies. The functional importance of circ_0009043 in NSCLC, however, has yet to be established.

**Methods:**

The expression of circ_0009043, miR-148a-3p, and DnaJ heat shock protein family (Hsp40) member B4 (DNAJB4) in NSCLC cells was assessed via qPCR. The proliferative activity of these cells was examined through EdU uptake and CCK-8 assays, while flow cytometry approaches were used to examine apoptotic cell death rates. Protein expression was measured through Western immunoblotting. Interactions between miR-148a-3p and circ_0009043 or DNAJB4 were detected through RNA immunoprecipitation (RIP) and dual-luciferase reporter assays. The *in vivo* importance of circ_0009043 as a regulator of oncogenic activity was assessed using murine xenograft models.

**Results:**

Both NSCLC cells and tissue samples were found to exhibit circ_0009043 upregulation, and lower circ_0009043 expression levels were found to be related to poorer NSCLC patient overall survival. Knocking down circ_0009043 resulted in the enhancement of NSCLC cell proliferative activity and the suppression of apoptotic tumor cell death *in vitro,* while also driving more rapid *in vivo* tumorigenesis. Mechanistically, circ_0009043 was found to function as a molecular sponge that sequestered miR-148a-3p, which was in turn able to directly suppress DNAJB4 expression. When miR-148a-3p was overexpressed, this reversed the impact of knocking down circ_0009043 on the apoptotic death and proliferation of NSCLC cells. Conversely, miR-148a-3p inhibition resulted in the suppression of NSCLC cell apoptosis and the enhancement of tumor cell growth, while the downregulation of DNAJB4 reversed these changes.

**Conclusion:**

Circ_0009043 acts as a tumor suppressor in NSCLC cells, promoting DNAJB4 upregulation via the sequestration of miR-148a-3p.

## Background

Lung cancer remains the deadliest form of cancer in the world, accounting for approximately 1.6 million deaths annually [1]. In 2020 alone, 228,820 new cases of lung cancer were reported in the USA together with 135,720 deaths [2]. Non-small cell lung cancer (NSCLC) is the most prevalent lung cancer histological subtype, making up ~ 85% of these cases. The 5-year survival rate for NSCLC patients is poor at just 19%, in part as many patients are diagnosed when the disease is relatively advanced and late-stage treatments for this cancer are lacking [3, 4]. The diagnosis and treatment of NSCLC are challenging owing to the high degree of neoplastic diversity within tumors and the challenges inherent in collecting the volumes of tissue necessary for reliable pathological or molecular diagnostic analyses and characterization [5, 6]. Genetic changes in tumors can also shift the associated molecular marker profiles over the course of disease. Biopsy or cytology samples are the primary materials used to diagnose most patients with advanced NSCLC at present, as resected tumor tissue availability is limited [7]. As such, there is a clear need for the identification of more reliable biomarkers of NSCLC and the further characterization of the mechanisms governing the pathogenesis of this disease so as to better guide the diagnosis and clinical treatment of patients such that the most effective surgical and therapeutic interventions can be administered to improve survival outcomes.

Circular RNAs (circRNAs) are a recently identified group of covalently closed non-coding RNAs formed through reverse splicing of their 3′ and 5′ ends [8]. Owing to their structural characteristics, circRNAs are very stable, in addition to being abundantly expressed and highly conserved. Indeed, circRNAs, which can be derived from intergenic, exonic, or intronic regions of the genome, often exhibit tissue- and developmental stage-specific patterns of expression [8, 9]. Functionally, these circRNAs can serve as scaffolds for protein complexes such that they can regulate the expression of genes including the parental genes from which they are derived. Moreover, they can influence alternative splicing events, modulate interactions between RNAs and proteins, and function as molecular sponges to sequester microRNAs (miRNAs) in a sequence-specific manner [10]. A growing body of evidence suggests that circRNAs are critical regulators of many forms of cancer including thyroid cancer, osteosarcoma, breast cancer, pancreatic cancer, hepatocellular carcinoma, and gastric cancer wherein they can act in an oncogenic manner, offering value as promising clinical biomarkers and/or viable targets for therapeutic intervention [11–15]. The recently identified circRNA_0009043 species of circRNA has been reported to be significantly downregulated in grade 1–3 endometrial cancer tissues relative to normal paracancerous endometrial tissue [16]. The functional importance of this circRNA in NSCLC, however, has yet to be defined.

The present study was developed to explore the relationship between circRNA_0009043 and NSCLC pathogenesis *in vitro* and *in vivo.* Functionally, this circRNA was found to inhibit NSCLC cell proliferation while promoting the apoptotic death of these cells. Mechanistically, circRNA_0009043 was found to regulate the miR-148a-3p / DNAJB4 axis so as to suppress NSCLC development.

## Methods

### Tissue specimens

All specimens were obtained from the Department of Thoracic Surgery, Hunan Provincial People’s Hospital. A total of 132 pairs NSCLC tissues and compared normal tissues were confirmed pathologically. More specifically, tumor and adjacent normal tissues were obtained from the patients who were diagnosed with NSCLC and underwent initial surgery between September 2014 and June 2016. These patients had not received any systemic treatment, including radio-chemical therapy, before sampling. The protocol was approved by the ethical review committee of Hunan Provincial People’s Hospital, and patient consent was obtained before the samples were taken.

### Cell line culture and transfection

The human NSCLC cell lines (A549 cells and HCC827 cells) were provided by the American Type Culture Collection (ATCC, Rockville, MD). Cells received incubation treatment in RPMI-1640 (Solarbio, Beijing, China) and dulbecco’s modified eagle medium (DMEM; Solarbio) composed of 1% penicillin/streptomycin (Solarbio) and 10% fetal bovine serum (FBS; Solarbio) under 5% CO_2_ at 37 °C. A pcircRNA2.2-circ_0009043 overexpression plasmid and empty pcircRNA2.2 plasmid, the pcDNA 3.1 plasmid specific to DNAJB4 (termed OE- DNAJB4) and empty pcDNA3.1 plasmid were purchased from Guangzhou RiBoBio Co., Ltd. (Guangzhou, China). Circ_0009034 short hairpin (sh) RNA (shcirc) (AATTCAAAAAACCATGAAGCAAAATCAAGTGATCTCTTGAAGTAGCACAACATTCTCCACCCG), scrambled shRNA (shCTRL), DNAJB4 short hairpin (sh) RNA (shDNAJB4) (AATTCAAAAAACAGAAGCTTATGAAGTATTGAGTTCTCTTGAAGTAGCACAACATTCTCCACCCG), miR-148a-3p mimics, NC miRNA mimic (mimic NC), miR-148a-3p inhibitor, and inhibitor NC were designed and synthesized by GenePharma Co., Ltd. (Shanghai, China). Lipofectamine 2000 (Invitrogen, Carlsbad, CA, USA) was employed to transfect abovementioned plasmids into A549 cells and HCC827 cells. 48 h after the transfection, we harvested cells for later study.

### Cell proliferation experiment

The proliferation of HCC cells was tested through Cell Counting Kit-8 (CCK-8) assay, the transfected A549 and HCC827 cells (1 × 10^4^ cells/well) were added into 96-well plates and maintained for 48 h. Next, 10 μL CCK-8 reagent (Sigma-Aldrich) was added into each well and cultured for further 2 h. After that, a microplate reader was adopted to examine the optical density value at 450 nm.

Cell proliferation was also assessed using an EDU assay kit (Ribobio, Guangzhou, China). For this purpose, after seeding 1 × 10^6^ cells into each well of confocal plates, a 2-h incubation was performed at 37 °C with 50 μM of EDU buffer. Cell fixing was then carried out for half an hour using 4% of formaldehyde before cell permeabilization for 20 min using 0.1% Triton X-100. Eventually, after adding the EDU solution, cell nuclei were stained with Hoechst prior to visualization under a fluorescence microscope.

### *In vivo *xenograft experiments

Nude female mice from Vital River Laboratories (Beijing, China) were housed under specific pathogen-free conditions in the Department of Laboratory Animal Science of Hunan Provincial People’s Hospital. The Ethics Review Committee of the Department of Laboratory Animal Science of Hunan Provincial People’s Hospital approved this study. All animals were randomly assigned to five groups (*n* = 6/group) that were then subcutaneously implanted in the left flank with A549 cells (1 × 10^7^) that were either WT cells or cells that had been stably transfected with the pcircRNA2.2, pcircRNA2.2-circ_0009043 vector, NC‑shRNA, or circ_0009043‑shRNA constructs. Tumor volume was measured once per week using calipers (V = length × width^2^ × 0.5) for four weeks, after which mice were euthanized. Tumors were then excised, fixed for 24 h using 4% paraformaldehyde, stained via TUNEL assay, and subjected to downstream immunohistochemical staining for DNAJB4, followed by imaging with a brightfield microscope (Olympus, Tokyo, Japan).

### Quantitative real-time reverse transcription PCR

Tissue samples and cells were collected, and total RNA was extracted using the TRIzol reagent (Invitrogen, Waltham, MA, USA) for quantitative real-time reverse transcription PCR (qRT-PCR) detection. The cDNA obtained by reverse transcription using the Promega GoScript reverse transcription system (Promega Madison, WI, USA) was subjected to quantitative real-time PCR in a 20 µL PCR amplification system using the ABI7500 real-time quantitative PCR instrument. For miRNAs, miRNA first-strand cDNA was constructed using the stem-loop method [17] using the cDNA Synthesis Kit (R601, Novabio, Shanghai, China). Stem-loop primer 5’-CTCAACTGGTGTCGTGGAGTCGGCAATTCAGTTGAGACAAAGTT-3’ was purchased from General Biol (Anhui, China). The differences in the expression levels of different genes were analysed using 2^−ΔΔCt^ relative quantification. The primers were circ_0009043: (F: 5’-TCCGCAAACATTCAGACAAA-3’ and R: 5’-GCTTCAGCTCTTCCATTGCT-3’); miR-148a-3p: (F: 5’-ACACTCCAGCTGGGTCAGTGCACTACAGAACT-3’ and R: 5’-CTCAACTGGTGTCGTGGA-3’); DNAJB4: (F: 5’-TGGGGACGCTGTTTTCTTTTAC-3’ and R: 5’-CTCCTGCTCCTCCTTTCAACC-3’); U6: (F: 5’-CTCGCTTCGGCAGCACA-3’ and R: 5’-AACGCTTCACGAATTTGCGT-3’); and GAPDH: (F: 5’-CTCCTCCTGTTCGACAGTCAGC-3’ and R: 5’-CCCAATACGACCAAATCCGTT-3’).

### Western immunoblotting

After isolation from tissue or cell samples, total proteins were separated via SDS-PAGE and transferred onto PVDF membranes that were blocked with an appropriate solution for 1 h at room temperature. Blots were then probed for 2 h at room temperature with primary antibodies specific for Bax (1:1000, BA0315-2, Boster), Bcl-2 (1:1200, A00040-1, Boster), Cytochrome C (1:5000, ab133504, Abcam), DNAJB4 (0.1 µg/ml, ab254641, Abcam), and GAPDH (1:10,000, BA2913, Boster). Blots were then probed for 1 h at room temperature using Goat Anti-Rabbit IgG H&L (HRP) (1: 2000, ab6721, Abcam), after which they were washed, incubated in developer solution, and exposed to X-ray film to detect protein bands.

### Subcellular fractionation

Nuclear and cytoplasmic fractions from A549 and HCC827 cells were isolated using a PARIS Kit (Invitrogen) based on provided directions. Circ_0009043 abundance in these two fractions was assessed via qPCR, with U6 and GAPDH being used as respective normalization controls for nuclear and cytoplasmic transcripts.

### Dual-luciferase reporter gene assay

Potential miRNA binding partners for circ_0009043 were predicted using an online tool (https://circinteractome.nia.nih.gov/). Luciferase reporter constructs were then prepared for circ_0009043 and miRNAs of interest, with HEK293T cells being transfected using WT or mutant circ_0009043 reporter constructs and transfected with appropriate miRNA mimic or control constructs. At 48 h post-transfection, firefly and Renilla luciferase activity was assessed, with results being reported as the ratio of these two activity levels.

### RNA immunoprecipitation (RIP)

A Magna RIP RNA‐Binding Protein Immunoprecipitation Kit (Millipore, MA, USA) was used based on provided directions. Total RNA and species-appropriate IgG (*n* = 3) were utilized as control samples to confirm the specificity of the detected RNA signal. For the anti‐AGO2 RIP assay, NSCLC cells were transfected using miRNA mimics after which RIP was performed using anti-AGO2 (2 µg/mL, clone number: ab32381; Abcam) at 48 h post-transfection.

### Flow Cytometry

After incubation for 48 h, cells that have been transfected and were seeded for the counting in the 96-well plates were then removed from the plates by using trypsin reagent in the absence of ethylenediaminetetraacetic acid, and cells were then rinsed with cooled PBS solution. After that, centrifugation was done, and the PBS solution was discarded. Then annexin V–fluorescein isothiocyanate (FITC) apoptosis discovery kit (BioLegend, Inc.) was applied to detect the status apoptotic of the obtained cells. Resuspension of cells was then carried out in 100 μL of buffer (1 × binding buffer). Then resuspended cells were further treated with 5 μL propidium iodide and annexin V–FITC in the darkness for the 15-min duration, and at room temperature. Finally, for the analysis of the double-stained cells, the FACSCalibur Flow Cytometry instrument (BD Biosciences, San Jose, CA, USA) was used. Version 2.9 of the CellQuest (BD Biosciences) was used for the evaluation of the proportion of apoptotic cells.

### Immunohistochemical (IHC) staining

Tissue sections were deparaffinized, subjected to antigen retrieval, treated to quench endogenous peroxidase activity, blocked using goat serum (Gibco), and incubated with primary anti-DNAJB4 (1 µg/ml, ab254641, Abcam) and secondary antibodies (abcam; #ab205718; 1:1000). Sections were then imaged using a phase-contrast microscope (Leica, Cat. #DMI 1).

### Terminal deoxynucleotidyl transferase-medimed dUTP nick-endlabeling (TUNEL) assay

A one-step TUNEL kit (C1089, Beyotime Institute) was used as in prior reports to assess apoptotic cell death. Briefly, NSCLC tissue Sect. (3 μm) were deparaffinized, treated for 30 min with DNase- protease K (20 μg/mL) at 37℃, rinsed using PBS, and stained in the dark for 1 h with 50 μL of TUNEL reaction mixture at 37℃. Sections were then rinsed using PBS and imaged with a fluorescence confocal microscope (Zeiss LSM710, Germany). TUNEL-positive cells in 10 random fields of view in size sections were then counted using Image J (Bio-Rad Laboratories, CA, USA).

### Statistical analysis

SPSS 18.0 (SPSS, Inc.) was used to analyze all data. Data were compared between groups using t-tests and one-way ANOVAs with Dunnett’s post hoc test as appropriate. Pearson’s correlation coefficient was used to assess the relationships between circ_0009043, miR-148a-3p, and DNAJB4 expression. Data are given as means ± SD from triplicate experiments, with P < 0.05 as the significance threshold.

## Results

### Circ_0009043 is downregulated in NSCLC and predicted a poor prognosis

**Fig. 1:**
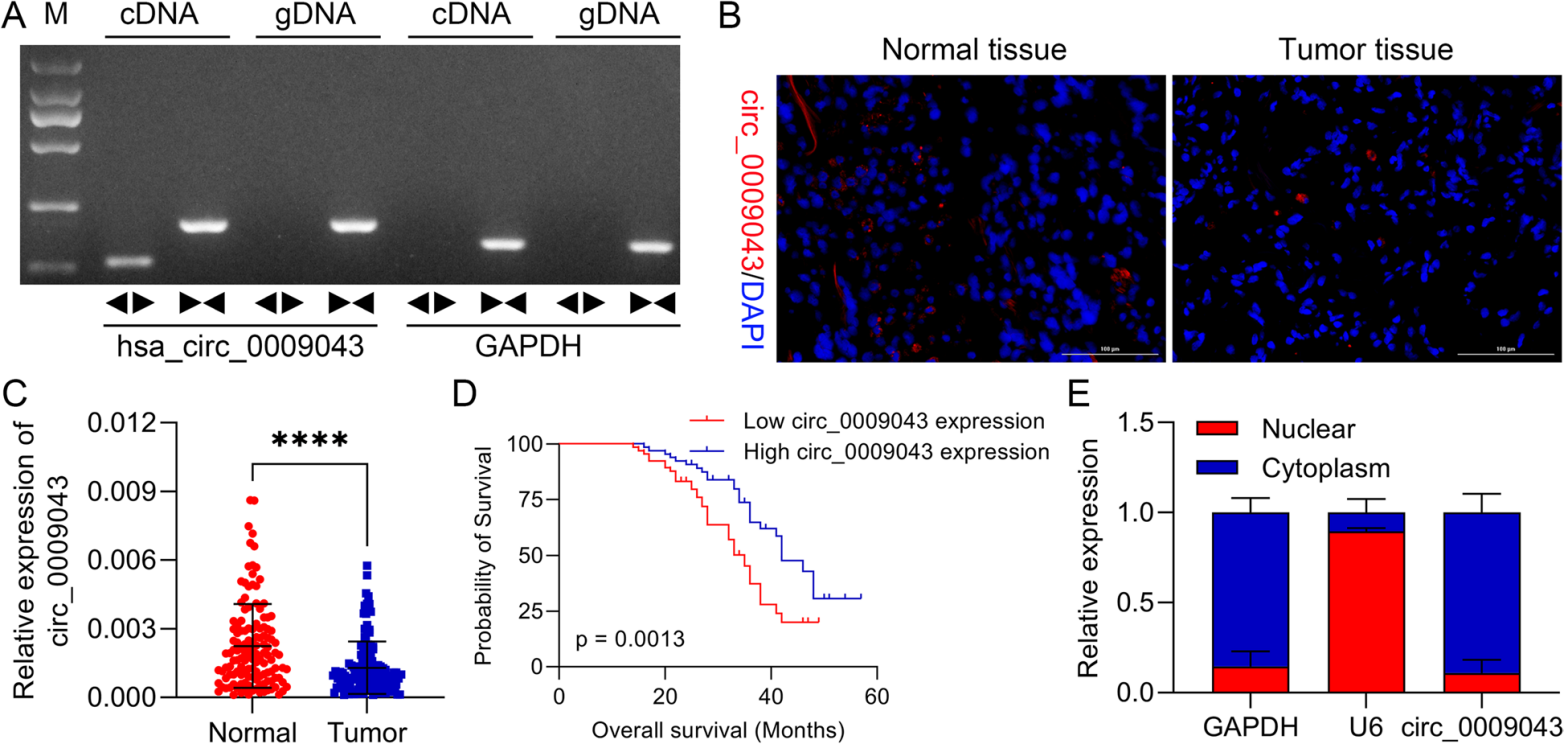
Downregulated circ_0009043 in NSCLC is associated with poor prognosis. **A** PCR product of cric_0009043 was detected in 1.5% agarose gel electrophoresis. **B** Representative images of circ_0009043 determined by RNA-FISH in NSCLC and normal tissues (× 200, scale bar 100 μm), respectively. **C** The expression level of circ_0009043 in that of NSCLC tissues along with normal lung tissues, *n* = 132. **D** Kaplan–Meier curve was plotted for the analyses of patients' survival rate on the ground of circ_0009043 expression. **E** Cellular localization of circ_0009043 in NSCLC cells was identified by employing RNA FISH while fractions of cytoplasmic and nuclear RNA were isolated and measured by performing RT-qPCR analysis**.** All results were representative of three separate experiments. Data represent mean values ± SD from three replicates of each sample; ****P < 0.0001

As shown in Fig. 1A, circular properties of the circ_0009043 were recognized through divergent primers as well as convergent primers. Then, we assessed its expression in tissue sections from NSCLC patients by FISH. Intriguingly, circ_0009043 was downregulated in the NSCLC as compared to the tissues of the normal lung (Fig. 1B). Moreover, the expression of circ_0009043 in the NSCLC tissues and in paired normal tissues was examined applying qRT-PCR approach. The results displayed that circ_0009043 level was downregulated significantly in the lung cancer tissues than in the adjacent nontumor tissues (Fig. 1C). Then, the 132 NSCLC tissues were separated into two sub-groups based upon circ_0009043 expression (the median value as cut-off). Kaplan–Meier curve was plotted for the analysis of the survival rate. The results indicated that circ_0009043 lower group of ovarian patients have a shorter survival rate (Fig. 1D). Moreover, the clinical relevance of this was then evaluated, showing that LINC00326 levels were linked with TNM stage, tumor differentiation, and lymphatic metastasis in NSCLC patients (Table 1). These results specified that circ_0009043 is clearly downregulated in NSCLC and predicted a poor prognosis. Afterwards, the cytoplasm and nucleus segmentation and RNA-FISH analyses verified that the circ_0009043 was predominantly localized in cell cytoplasm instead of their nucleus. RT-qPCR was performed to analyze fractions of the cytoplasmic as well as nuclear RNA in order to ascertain the circ_0009043 localization in the NSCLC cells. The obtained findings displayed that the circ_0009043 was predominantly detected within the cytoplasm of NSCLC cells (Fig. 1E), indicating that circ_0009043 had a primary effect on the cytoplasm of NSCLC cells.

**Table 1:** Correlation between circ_0009043 expression and clinicopathological features in 132 NSCLC patients

Parameters	Group	N	Expression of circ_0009043		*P* value
			Low, n (%)	High, n (%)	
Age (years)	≤ 56	62	34 (54.84)	28 (45.16)	0.7901
	> 56	70	40 (57.14)	30 (42.86)	
Gender	Female	64	29 (45.31)	35 (54.69)	0.8902
	Male	68	30 (44.12)	38 (55.88)	
Tumor size (cm)	< 3	61	35 (57.38)	26 (42.62)	0.1585
	≥ 3	71	32 (45.04)	39 (54.93)	
TNM stage	I–II	63	23 (36.51)	40 (63.49)	**0.0018**
	III–IV	69	44 (63.77)	25 (36.23)	
Smoking history	Yes	62	29 (46.77)	33 (53.23)	0.3050
	No	70	39 (55.71)	31 (44.29)	
Lymph node metastasis	Yes	95	62 (65.26)	33 (34.74)	**0.0097**
	No	37	15 (40.54)	22 (59.46)	
Differentiation	Well/moderate	62	20(32.26)	42(67.74)	**0.0042**
	Poor	70	40(57.14)	30(42.86)	

*NSCLC* Non-small cell lung cancer

### Overexpression of circ_0009043 inhibits the proliferation, while accelerating apoptosis of the NSCLC cells

**Fig. 2:**
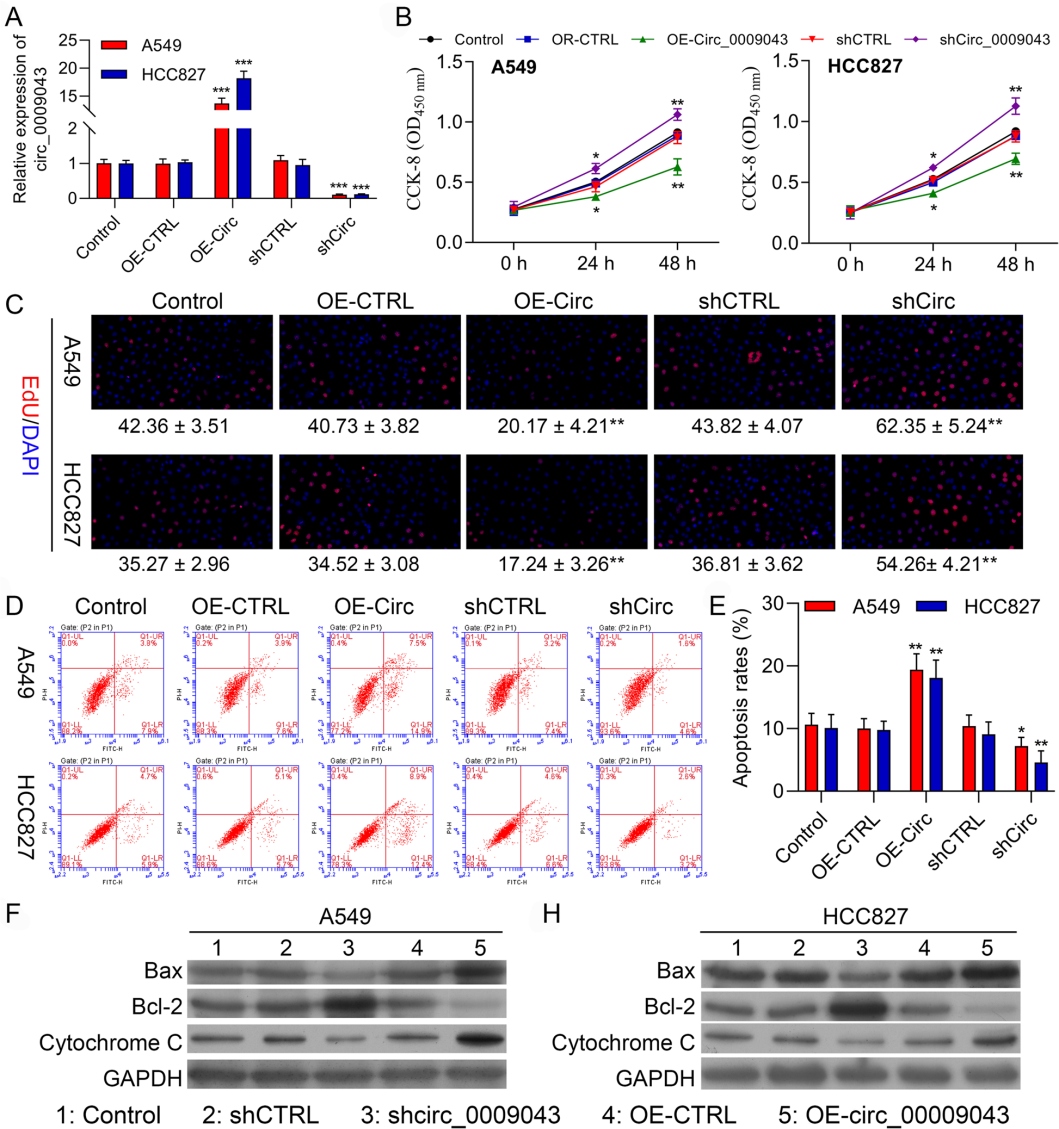
Overexpression of circ_0009043 inhibits the proliferation, while accelerates apoptosis of NSCLC cells. **A** circ_0009043 mRNA expression in HCC827 and A549 cells transfected with pcDNA4.0 vector (OE-CTRL), pcDNA4.0- circ_0009043 vector (OE-Circ), CTRL‑shRNA (shCTRL), or circ_0009043‑shRNA (shCirc) were determined by RT-qPCR. **B** CCK8 assay was used to compare the cell proliferation of Control, OE-CTRL, OE-Circ, shCTRL, and shCirc groups in HCC827 and A549 cells. **C** Edu assay of Control, OE-CTRL, OE-Circ, shCTRL, and shCirc groups in HCC827 and A549 cells. **D-E** The ratio of apoptosis in the HCC827 and A549 cells transfected with indicated vectors, which consisting of the OE-CTRL, OE- Circ, shCTRL and shCirc groups were detected by flow cytometry. Comparison of the ratio of apoptosis in the afore mentioned 5 groups. Each bar indicates the mean apoptosis rate ± standard deviation per group. **F-G** Expression level of Bax, Bcl-2 and Cytochrome C in the HCC827 and A549 cells of the 5 groups (CTRL, OE-CTRL, OE- Circ, shCTRL and shCirc groups) were determined by western blotting. Data was normalized to GAPDH. All results were representative of three separate experiments. Data represent mean values ± SD from three replicates of each sample; *P < 0.05, **P < 0.01, ***P < 0.001

First, we found circ_0009043 was expressed highest in HCC827 cell line and lowest A549 cell line in different NSCLC cell lines, including A549, HCC827, HCI-H1299, NCI-H1568, NCI-H1650, and HCI-H358 (Data not shown). Thus, we selected HCC827 cell line and A549 cell line for further studies. The transfection efficacies of the circ_0009043 overexpression vector and shRNA targeting circ_0009043 were validated through the qRT-PCR in the A549 and HCC827 cells (Fig. 2A). After this, the CCK-8 assay was performed for determining the effect of circ_0009043 on cell proliferation. Figure 2B represents that the cell proliferation was remarkably inhibited in the circ_0009043 overexpression group than that of the negative control group (OE-CTRL) in the A549 as well as HCC827 cells. In the same way, circ_0009043 knockdown cells get proliferated faster as compared to the shCTRL group. In parallel, as presented in Fig. 2C, the Edu assay showed significantly less positive cells after circ_0009043 overexpression in A549 and in HCC827 cells. These obtained results indicate that circ_0009043 suppressed the NSCLC cells proliferation. Apoptosis is a key contributing factor that regulates the malignancy in the NSCLC. For investigating the cancer suppressor activity of circ_0009043 through apoptosis regulation, we scrutinized the apoptosis through FCM assay. The flow cytometry (FC) results displayed that the rate of apoptosis was decreased in the shCirc_0009043 transfected cells, and increased in the circ_0009043 overexpression cells as observed in Fig. 2D-E. The data obtained revealed that circ_0009043 accelerates the *in vitro* apoptosis of NSCLC cells. Additionally, the expression levels of Bax and Cytochrome C were gets decreased in the A549 as well as HCC827 cells transfected with sh-circ_0009043 as compared to sh-CTRL groups. On the contrary, circ_0009043 upregulation led to an increased level of the Bcl-2 as compared to the control groups (Fig. 2F-G). Collectively, circ_0009043 exerted a tumour-suppressing role in NSCLC.

### Circ_0009043 functioned as a sponge for miR-148a-3p in NSCLC cells

**Fig. 3:**
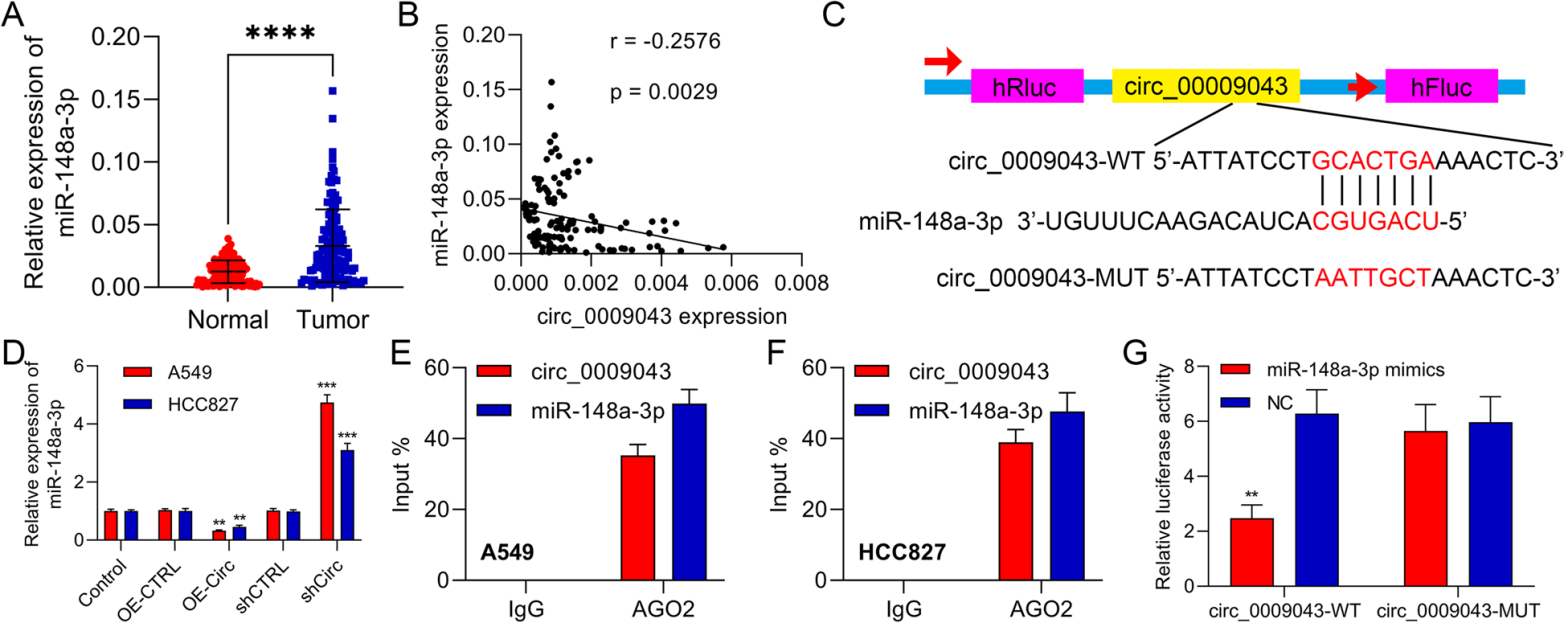
Circ_0009043 functioned as a sponge for miR-148a-3p in NSCLC cells. **A** RT-qPCR was performed for ascertaining the expression of miR-148a-3p in NSCLC tissues compared with normal lung tissues. **(B)** Correlations between circ_0009043 and miR-148a-3p. **C** Online bioinformatics analysis showed the binding within that of miR-148a-3p in addition to 3’-UTR of circ_0009043. **D** Expression level of miR-148a-3p in the HCC827 and A549 cells of the 5 groups (CTRL, OE-CTRL, OE- Circ, shCTRL and shCirc groups) were determined by RT-qPCR. **E–F** Determination of the physical interaction between circ_0009043 and miR-148a-3p by RNA immunoprecipitation experiments. **G** The activity of circ_0009043-wt plasmid in A549 cells was suppressed by the mimic transfection of miR-148a-3p which was demonstrated by luciferase reporter assay. ***P < 0.001; ****P < 0.0001

We then identified the miR-148a-3p expression in the NSCLC as compared with the tissues of normal lungs and observed that the miR-148a-3p was upregulated considerably in NSCLC tissues (Fig. 3A). Similarly, an inverse expression correlation was observed between circ_0009043 and miR-148a-3p in NSCLC tissues (Fig. 3B). Additionally, we observed that miR-148a-3p might target circ_0009043 by using the online prediction software Starbase (https://starbase.sysu.edu.cn/). The active binding site for the miR-148a-3p in the 3’UTR of circ_0009043 was recognized (Fig. 3C). Furthermore, the transfection of circ_0009043 overexpression vector downregulated miR-148a-3p level in the A549 as well as HCC827 cells. On the contrary, the transfection of shRNA targeting circ_0009043 upregulated miR-148a-3p level (Fig. 3D). RIP experiments displayed that the miR-148a-3p coprecipitated with circ_0009043, confirming the strong physical interaction between the circ_0009043 and miR-148a-3p (Fig. 3E-F). Moreover, Luciferase reporter assay represented that the miR-148a-3p overexpression suppressed the WT- circ_0009043 reporter activity (Fig. 3G). Therefore, these findings demonstrated that circ_0009043 establishes a direct connection with the miR-148a-3p and consequently, it mirrors the role of a molecular sponge for that of the miR-148a-3p in the NSCLC cells.

### MiR-148a-3p promotes proliferation, while inhibits apoptosis in the A549 and HCC827 cells

**Fig. 4:**
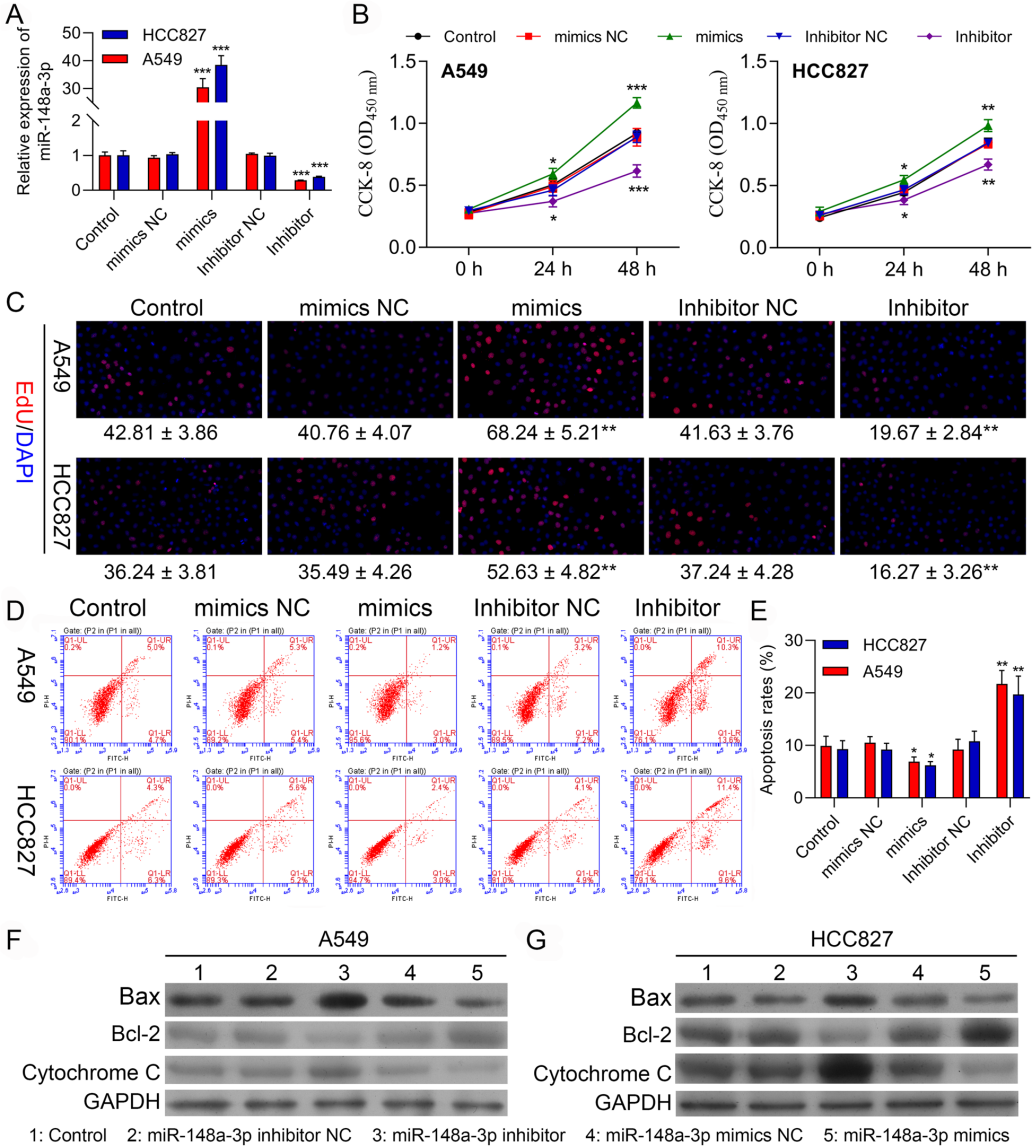
MiR-148a-3p promotes proliferation, while inhibits apoptosis in A549 and HCC827 cells. **A** qPCR assay confirming the transfection efficiency of the miR-148a-3p mimic and miR-148a-3p inhibitor in A549 and HCC827 cells. **B** Viability in A549 and HCC827 cells transfected with miR-NC, miR-148a-3p mimic, miR-148a-3p inhibitor NC, or miR-148a-3p inhibitor. **C** Edu assay of cell proliferation in A549 and HCC827 cells transfected with miR-NC, miR-148a-3p mimic, miR-148a-3p inhibitor NC, or miR-148a-3p inhibitor, respectively. **D-E** Cell apoptosis rates of A549 and HCC827 cells with the indicated transfection were determined with FSC assay. Data are presented as mean ± standard deviation. The experiments were repeated three times. **F-G** Protein levels of Bax, Bcl-2 and Cytochrome in A549 and HCC827 cells with the indicated transfection were determined by western blot. Data are presented as mean ± standard deviation. The experiments were repeated three times. * *P* < 0.05; ** *P* < 0.01; *** *P* < 0.001

We further evaluate the miR-148a-3p role in the A549 and HCC827 cells. The transfection efficacies of the miR-148a-3p inhibitor and miR-148a-3p mimic were examined in HCC827 and A549 cells as represented in Fig. 4A. Then, a CCK-8 assay was conducted for determining the influence of miR-148a-3p on the proliferation of the cells. The proliferation of cells was enhanced significantly in the miR-148a-3p overexpression group by comparing to the proliferation in the negative control group in the A549 and HCC827 cells, as observed in Fig. 4B. Likewise, cells transfected with the miR-148a-3p inhibitor proliferated slower comparatively than those transfected with inhibitor NC cells. Consistently, the EdU assay revealed that upregulation of the miR-148a-3p enhanced the NSCLC cells proliferation as observed in Fig. 4C. The obtained results clearly demonstrated that the miR-148a-3p promoted the NSCLC cells proliferation. Afterwards, we examined apoptosis via FCM assay. By comparing with the NC group, the results of FC displayed that the rate of apoptosis decreased in miR-148a-3p mimics transfected cells and increased in the miR-148a-3p downregulated cells (Fig. 4D-E). These data showed that miR-148a-3p prevented apoptosis of the NSCLC cells in vitro. At last, we also carried out the western blotting for investigating the changes in the expression of Bax, Bcl-2, and also Cytochrome C. The upregulated levels of protein of BAX and Cytochrome C induced by miR-148a-3p knockdown demonstrated substantial downregulation upon transfection with miR-148a-3p mimics (Fig. 4F-G). These results are further confirmed that miR-148a-3p acts as an oncogene in NSCLC.

### MiR-148a-3p Reversed the Regulatory Effect of circ_0009043 on A549 and HCC827 cells

**Fig. 5:**
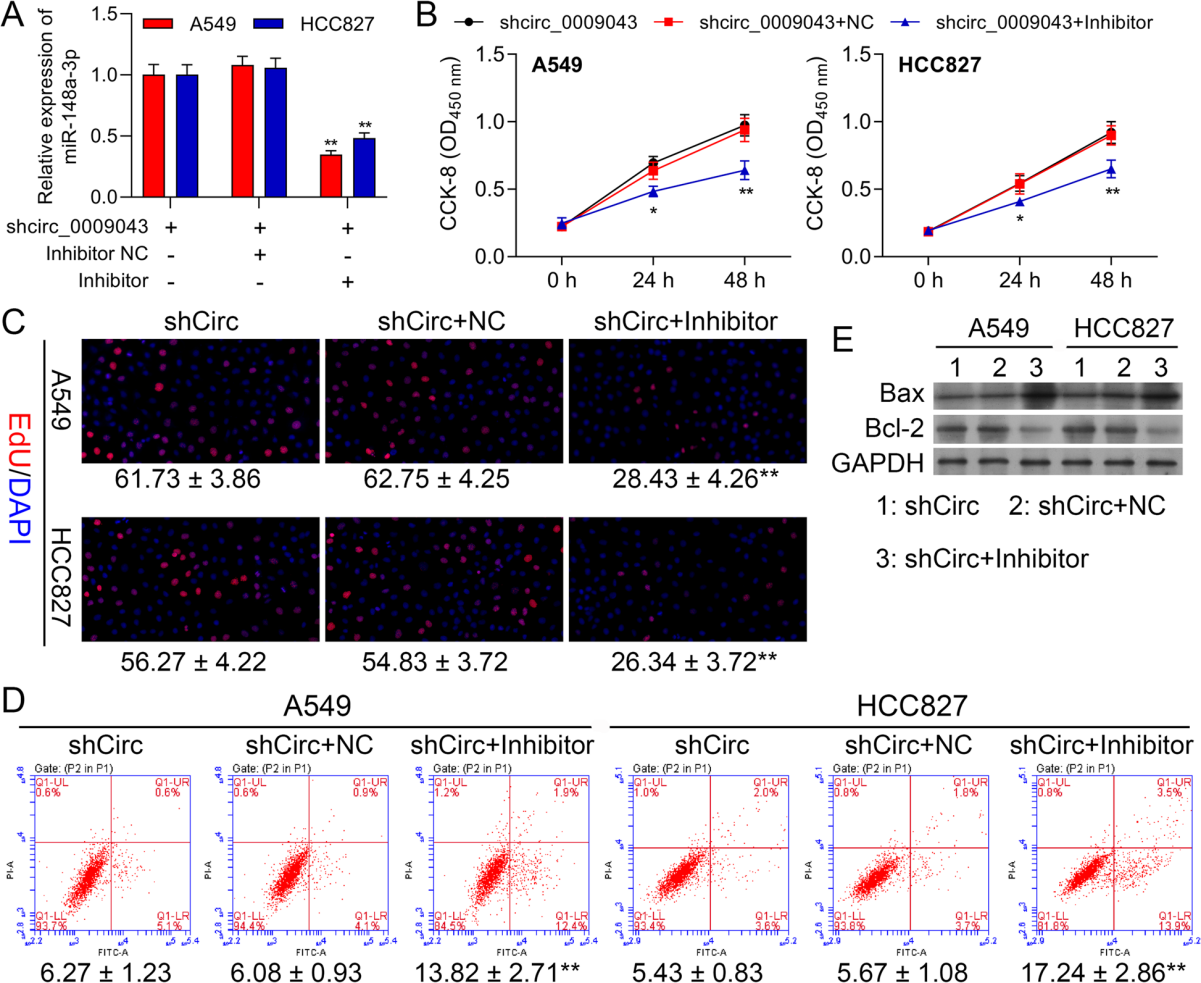
MiR-148a-3p Reversed the Regulatory Effect of circ_0009043 on A549 and HCC827 cells. A549 and HCC827 cells were transfected with shCirc, shCirc + NC, or shCirc + miR-148a-3p inhibitor. **(A)** MiR-148a-3p mRNA levels in A549 and HCC827 were determined with RT-qPCR assay. **B** Viability in A549 and HCC827 cells at 0, 24, and 48 h. **C** EdU assay to detect A549 and HCC827 cell proliferation. **(D)** FSC assay to detect A549 and HCC827 cell apoptosis. **E** Protein levels of Bax and Bcl-2 in A549 and HCC827 cells with the indicated transfection were determined by western blot. GAPDH is a loading control. Data are presented as mean ± standard deviation. **P < 0.01; *** *P* < 0.001

To clarify further the interaction of the circ_0009043 and miR-148a-3p, we performed carefully a series of rescue experiments. The upregulated level of the miR-148a-3p in A549 and HCC827 cells transfected shRNA targeting circ_0009043 was reversed through miR-148a-3p downregulation (Fig. 5A). Downregulation of circ_0009043 promoted the viability in A549 and HCC827 cells that was partially reversed via co-transfection of the miR-148a-3p inhibitor (Fig. 5B). Meanwhile, EdU assay showed that circ_0009043 downregulation mediated acceleration effect on the proliferation capability of the A549 and HCC827 cells get weakened after miR-148a-3p downregulated (Fig. 5C). Identically, the reduced apoptosis cell numbers in the A549, as well as HCC827 cells due to the downregulation of circ_0009043, were elevated after the co-transfection of the miR-148a-3p inhibitor (Fig. 5D). In addition, miR-148a-3p downregulation attenuated the shCirc facilitated upregulation of the E-cadherin, while downregulation of the N-cadherin, vimentin, and slug expression (Fig. 5E). Thus, it is believed that the downregulation of miR-148a-3p leads to reverse the regulatory effects of the circ_0009043 knockdown on proliferative and apoptosis abilities of NSCLC cells.

### DNAJB4 is a direct target gene of miR-148a-3p

**Fig. 6:**
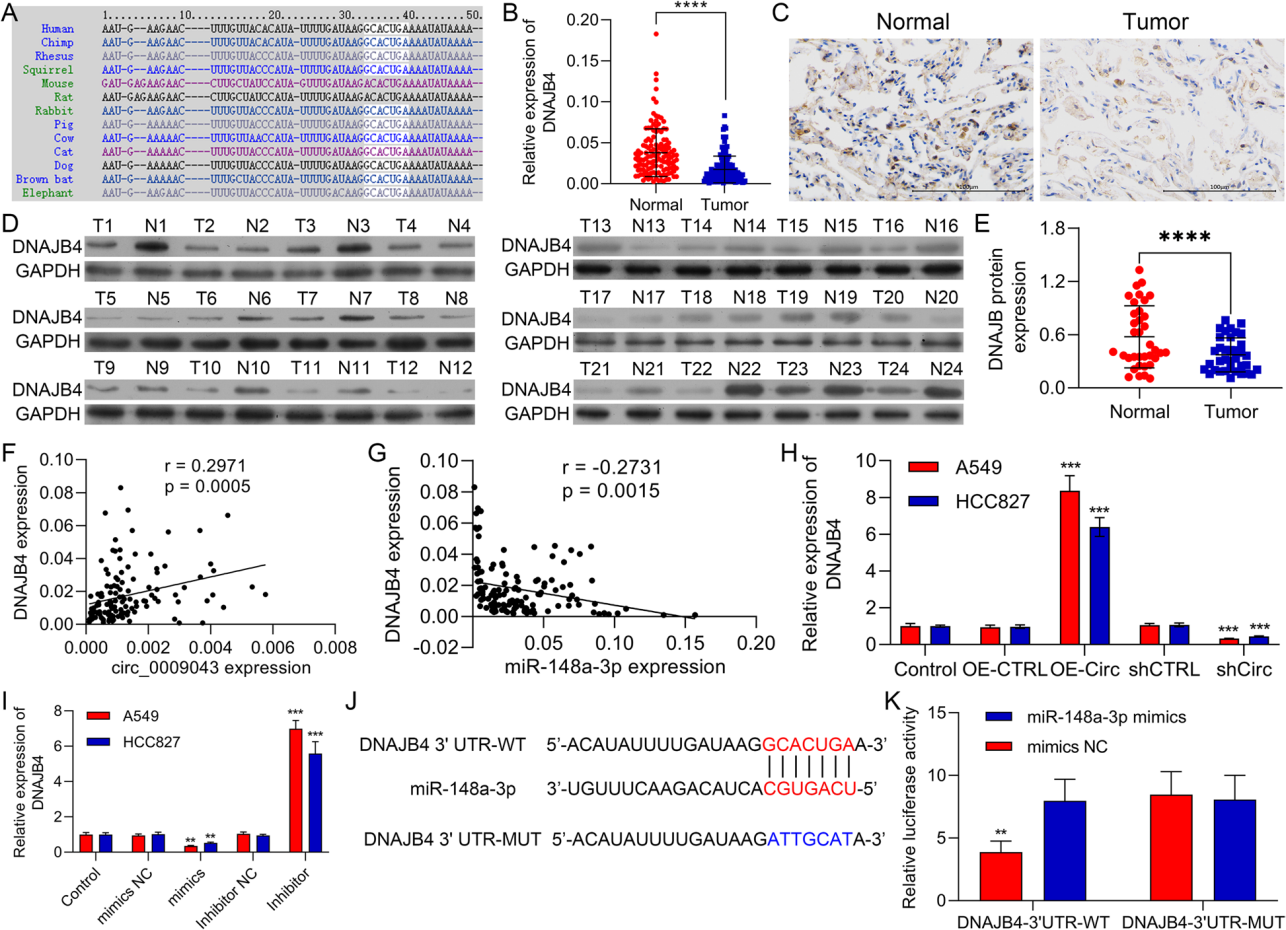
DNAJB4 is a direct target gene of miR-148a-3p. **A** DNAJB4 acted as a potential target of miR-148a-3p using bioinformatics analysis. **B** RT-qPCR was used to quantify DNAJB4 expression in NSCLC tissues as well as normal lung tissues, *n* = 132. **C** Representative images of circ_0009043 IHC staining of NSCLC and normal tissues (× 200, scale bar 100 μm), respectively. **D** The DNAJB4 protein expression level was significantly suppressed in NSCLC tissues compared with adjacent normal tissues in selected 24 paired tissues. **E** The expression level of DNAJB4 mRNA using RT-PCR in NSCLC and normal tissues (*n* = 24); **F** Correlations between DNAJB4 and circ_0009043. **G** Correlations between DNAJB4 and miR-148a-3p. **H** RT-qPCR analysis showed that circ_0009043 overexpression increased DNAJB4 level. **I** The mRNA level of DNAJB4 in A549 and HCC827 ells transfected with miR-148a-3p mimics or miR-148a-3p inhibitor was measured via RT-qPCR assay. **J** Online bioinformatics analysis showed the binding within that of miR-148a-3p in addition to 3’-UTR of DNAJB4. **K** The activity of DNAJB4-wt plasmid in NSCLC cells was suppressed by the mimic transfection of miR-148a-3p which was demonstrated by luciferase reporter assay. *P < 0.05, **P < 0.01, ***P < 0.001

For elucidating the fundamental mechanism of circ_0009043/miR-148a-3p axis in regulating the progression of NSCLC cells, we predicted the potential target genes for the miR-148a-3p through starBase. Consequently, we investigated the DNAJB4 as a potential target gene for the miR-148a-3p (Fig. 6A). Then, 66 tissues pairs of the clinical NSCLC and the matched adjacent were carefully chosen for detecting the expression levels of the circ_0009043 mRNA applying RT-PCR. Circ_0009043 mRNA expression in the NSCLC tissues was considerably downregulated than that observed in adjacent tissues (*P* < 0.0001; Fig. 6B), also recognized through IHC staining (Fig. 6C). Additionally, DNAJB4 protein in the NSCLC tissues was inspected using WB and it was detected that DNAJB4 expression was usually lower in the NSCLC tumor tissue than observed in adjacent tissue (Fig. 6D). The RT-qPCR assay was also carried out for detecting the expression level of DNAJB4 in NSCLC tissues as well as adjacent nontumor tissues (Normal). The obtained results demonstrated that the DNAJB4 level was noticeably decreased in NSCLC tissues as compared to Normal (Fig. 6E).

Similarly, an inverse expression correlation between DNAJB4 and the miR-148a-3p was notably detected, while DNAJB4 expression is a positive correlation with circ_0009043 (Fig. 6F-G). Then, we transfected circ_0009043 into A549 and HCC827 cells and clearly observed that circ_0009043 overexpression particularly enhanced the mRNA level of DNAJB4 in the A549 and HCC827 cells (Fig. 6H). Furthermore, miR-148a-3p mimic transfection significantly suppressed the level of DNAJB4 in the A549 as well as HCC827 cells (Fig. 6I), supporting miR-148a-3p targets DNAJB4. Also, we observed that the miR-148a-3p possibly targets DNAJB4 through bioinformatics investigation. Similarly, we recognized the active binding sites for the miR-148a-3p in 3’UTR of DNAJB4 (Fig. 6J). The Luciferase reporter assay specified that the miR-148a-3p overexpression suppressed the activity of the WT-DNAJB4 reporter (Fig. 6K). On the basis of the above results, it was speculated that DNAJB4 acts as a targeted gene of the miR-148a-3p.

### Overexpression of DNAJB4 inhibits the proliferation but promotes apoptosis of NSCLC cells

**Fig. 7:**
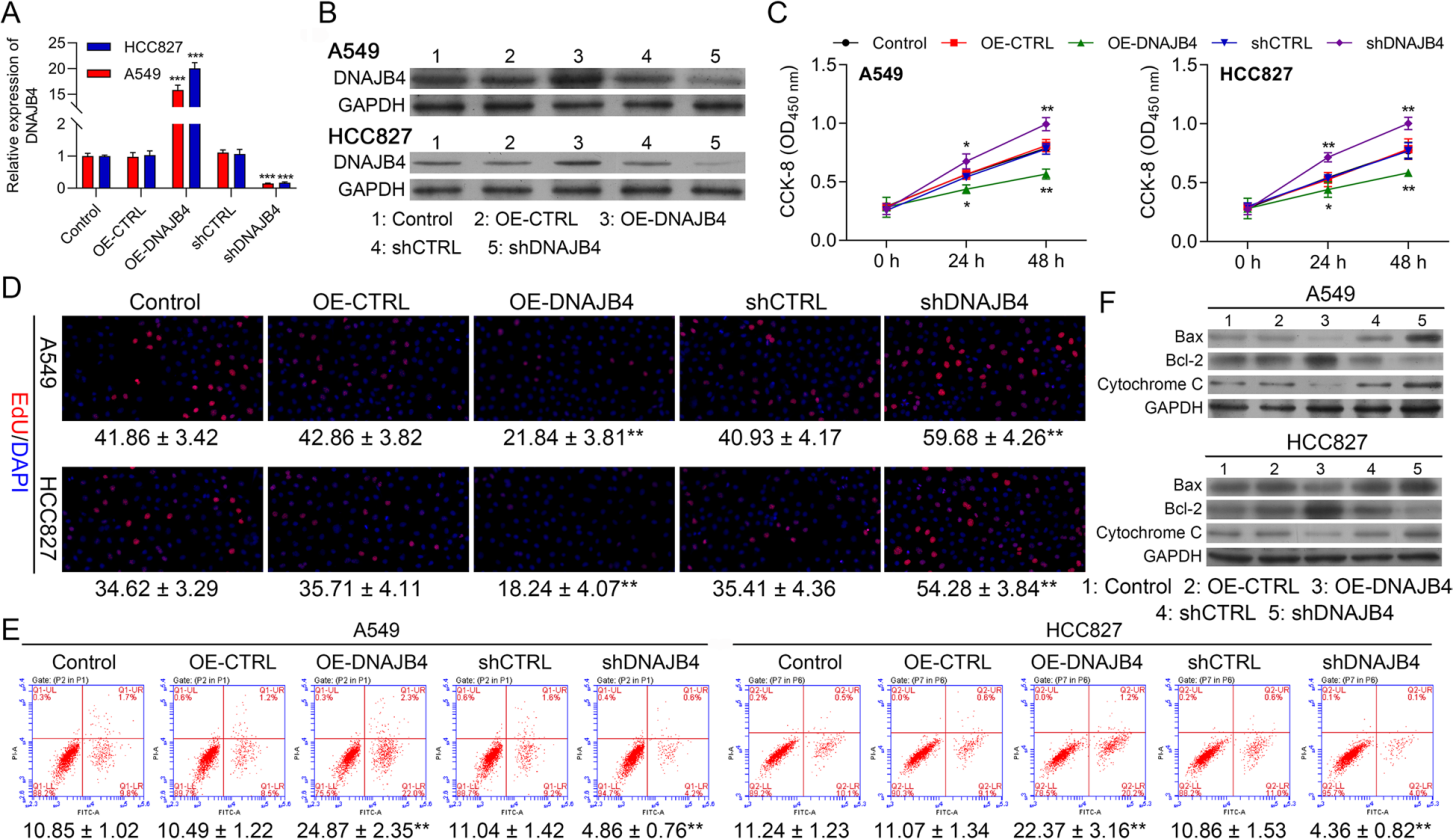
Overexpression of DNAJB4 inhibits the proliferation but promotes apoptosis abilities of NSCLC cells. **A** DNAJB4 mRNA expression in HCC827 and A549 cells transfected with pcDNA4.0 vector, pcDNA4.0- DNAJB4 vector, CTRL‑shRNA, or DNAJB4 ‑shRNA were determined by RT-qPCR. **B** DNAJB4 protein expression in HCC827 and A549 cells transfected with indicated vectors, which divided into five groups, including Control, OE-CTRL, OE-DNAJB4, shCTRL and shDNAJB4. **C** CCK8 assay was used to compare the cell proliferation of CTRL, OE-CTRL, OE- DNAJB4, shCTRL and sh DNAJB4 groups in HCC827 and A549 cells. **D** Edu assay of Control, OE-CTRL, OE- DNAJB4, shCTRL and sh DNAJB4 groups in HCC827 and A549 cells, respectively. **E** FSC assay to detect cell apoptosis of Control, OE-CTRL, OE- DNAJB4, shCTRL and sh DNAJB4 groups in HCC827 and A549 cells. **F** Expression level of Bax, Bcl-2 and Cytochrome C in the HCC827 and A549 cells of the 5 groups (Control, OE-CTRL, OE- DNAJB4, shCTRL and sh DNAJB4 groups) were determined by western blotting. Control negative control, OE over expression. Data represent mean values ± SD from three replicates of each sample; **P < 0.01, ***P < 0.001, ****P < 0.001

In the next step, we evaluate the DNAJB4 role in the NSCLC cells. RT-qPCR, as well as western blotting, revealed upregulation in the expression of DNAJB4 in NSCLC cells transfected with DNAJB4 overexpression vectors. In addition to that, sh DNAJB4 led to a decreased level of DNAJB4 as also confirmed through qRT-PCR as well as western blotting (Fig. 7A, B). Then, CCK8 and EdU assays were carried out for determining the effect of DNAJB4 on the proliferation of the cell. Figure 7C represents that the proliferation of cells was considerably suppressed in the DNAJB4 overexpression group as compared to observed in the negative control group (OE-CTRL) in A549 as well as HCC827 cells. Likewise, DNAJB4 -Knockdown proliferation of cells is faster as compared with the shCTRL group. Meanwhile, Edu positive numbers of A549, as well as HCC827 cells transfected with DNAJB4 overexpression vector (OE- DNAJB4), were considerably lower than those that are transfected with OE-CTRL. An opposite process was noticed in the sh DNAJB4 group (shDNAJB4) from the process observed in the shCTRL group in A549 as well as HCC827 cells (Fig. 7D). Moreover, by comparing with the OE-CTRL group, the FC results demonstrate that the rate of apoptosis gets increased in the cells transfected with DNAJB4 overexpression vectors while decreased in the DNAJB4 downregulated cells (Fig. 7E). In the last, we carried out the western blotting for determining the changes that occur in the expression of Bcl-2, Bax, and Cytochrome C. These findings showed that DNAJB4 downregulation led to the upregulation of Bcl-2, while downregulation of Bax, and Cytochrome C expression. However, opposite results can be observed by transfection of overexpression DNAJB4 vector in A549 and HCC827 cells (Fig. 7F). These obtained results collectively provided evidence that DNAJB4 inhibits the proliferation but promotes apoptosis of NSCLC cells.

### DNAJB4 Reversed the Regulatory Effect of miR-148a-3p on A549 and HCC827 cells

**Fig. 8:**
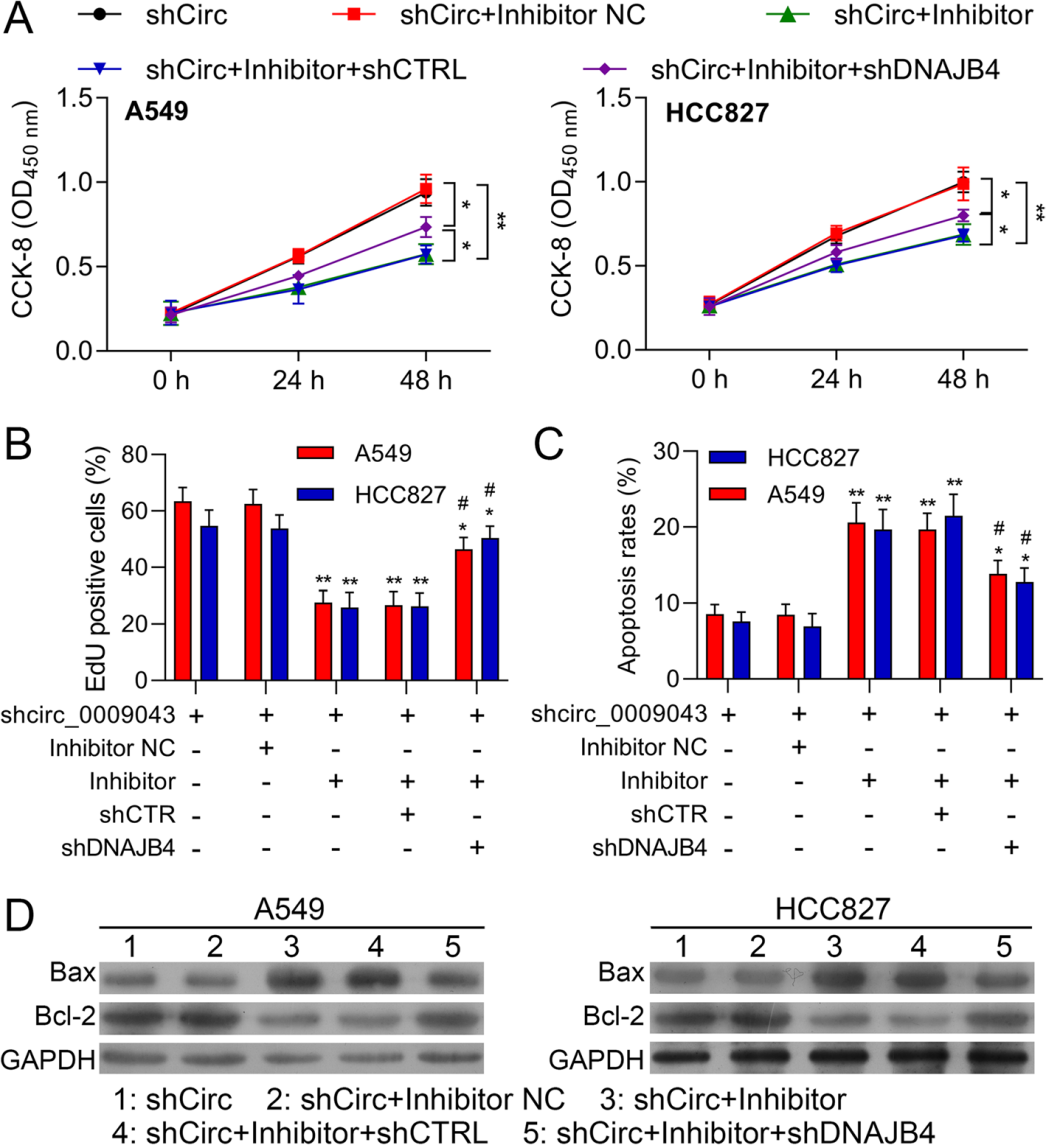
DNAJB4 Reversed the Regulatory Effect of miR-148a-3p on A549 and HCC827 cells. A549 and HCC827 cells were transfected with shCirc, shCirc + miR-148a-3p inhibitor negative control (Inhibitor NC), shCirc + miR-148a-3p inhibitor (Inhibitor), shCirc + miR-148a-3p inhibitor (Inhibitor) + shCTRL, or shCirc + miR-148a-3p inhibitor + shDNAJB4, respectively. **A** Viability in A549 and HCC827 cells at 0, 24, and 48 h. **B** EdU assay to detect A549 and HCC827 cell proliferation ability. **C** FSCS assay to detect A549 and HCC827 cell apoptosis. **D** Protein levels of E-cadherin, N-cadherin, Vimentin and Slug in A549 and HCC827 cells with the indicated transfection were determined by western blot. GAPDH is a loading control. Data are presented as mean ± standard deviation. **P < 0.01; *** *P* < 0.001

For exposing further the contact between circ_0009043, miR-148a-3p and DNAJB4, a series of rescue experiments were conducted. The transfection of the miR-148a-3p inhibitor reduced the viability in the A549 and NCI-HCC827 cells which were partially reversed by the co-transfection of shDNAJB4 (Fig. 8A). Meanwhile, the EdU assay displayed that the miR-148a-3p inhibitor facilitated the inhibition effect on the cell proliferation of the A549 and HCC827 cells were weakened after DNAJB4 downregulated (Fig. 8B). Identically, the increased apoptosis cell numbers in the A549, as well as HCC827 cells due to the transfection of the miR-148a-3p inhibitor, were suppressed after the knockdown of DNAJB4 (Fig. 8C). Moreover, DNAJB4 downregulation attenuated the miR-148a-3p inhibitor mediated upregulation of Bax, while downregulation of Bcl-2 expression (Fig. 8D). Thus, it is believed that the downregulation of DNAJB4 reversed the regulatory effects of the miR-148a-3p inhibitor on the proliferative and apoptosis abilities of NSCLC cells.

### Circ_0009043 inhibits tumor growth via targeting the miR-148a-3p / DNAJB4 pathway *in vivo*

**Fig. 9:**
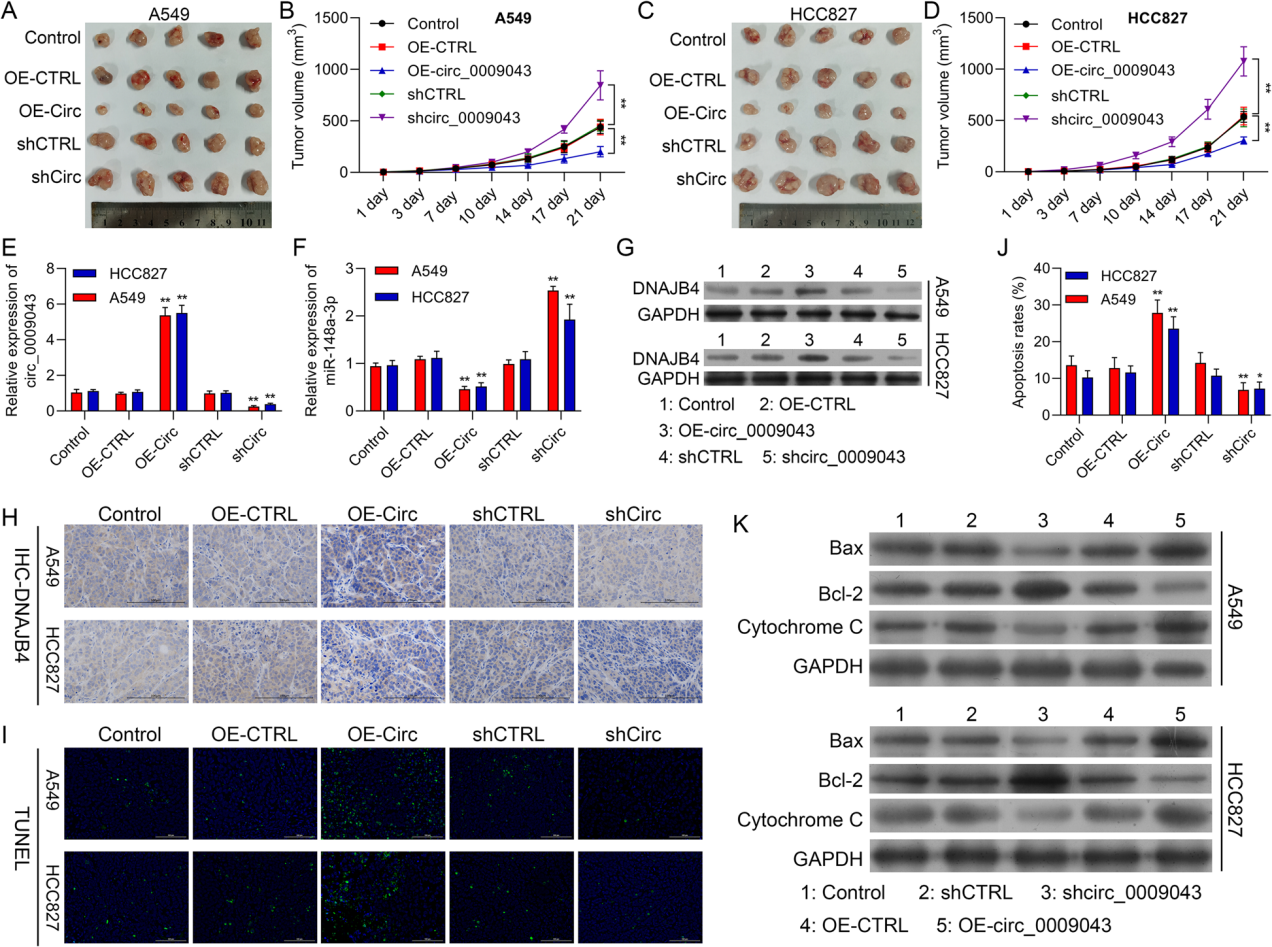
Circ_0009043 inhibits tumor growth via targeting the miR-148a-3p / DNAJB4 pathway in vivo. **A, C** Tumors formed 6 weeks post-injection in BALB/C nude mice. Tumors in the CTRL, OE-CTRL, OE-Circ, shCTRL and shCirc groups were isolated from mice at the endpoint of experiments. **B, D** Tumor growth was assessed by tumor volume measurement over time in the 5 afore mentioned groups (mean ± SD; *n* = 5). **P < 0.01. Mice were anesthetized and sacrificed at experimental endpoints. Tumors were subsequently dissected. **E, F** Circ_0009043 and miR-148a-3p mRNA expression in tumors from CTRL, OE-CTRL, OE-Circ, shCTRL and shCirc groups, respectively; *n* = 5. **G** DNAJB4 protein expression in tumors from CTRL, OE-CTRL, OE-Circ, shCTRL and shCirc groups; *n* = 5. **H** Representative images of DNAJB4 IHC in CTRL, OE-CTRL, OE-Circ, shCTRL and shCirc groups, respectively. (× 200, scale bars, 100 µm). **I-J** TUNEL staining assay was applied to compare the cell apoptosis of CTRL, OE-CTRL, OE-Circ, shCTRL and shCirc groups in tumors (scale bar, 100 μm). **K** Expression level of Bax, Bcl-2, and Cytochrome C in the cells of the 5 groups (CTRL, OE-CTRL, OE-Circ, shCTRL and shCirc groups) were determined by western blotting. **P < 0.01, ***P < 0.001, ###*P* < 0.001; ####*P* < 0.0001

We applied finally the xenograft tumor model for investigating the influence of circ_0009043 on tumor growth *in vivo*. pcDNA4.0 vector, pcDNA4.0- circ_0009043 vector, shCTRL, or shCirc transfected A549 or the HCC827 cells were injected subcutaneously in the left flank of the athymic nude mice, respectively. Like the previous cell experiments results, the obtained result suggested that the tumors of the circ_0009043 knockdown group (shCirc) were considerably bigger than that of the shCTRL group (*P* < 0.0001), while the tumors of circ_0009043 overexpression group (OE-Circ) were considerably smaller from that of the negative control group (OE-CTRL, *P* < 0.01) as depicted in Fig. 9A-D. Meanwhile, circ_0009043 mRNA expression in the 5 tumor groups established through differently treated cells was investigated through RT-PCR. These results demonstrated that the expression of the circ_0009043 was significantly enhanced by OE-Circ transfection while downregulated with shCirc transfection (Fig. 9E). However, the transfection of circ_0009043 overexpression vectors downregulated miR-148a-3p level in tumor the tissues. On the contrary, the transfection of shCirc_0009043 upregulated miR-148a-3p level (Fig. 9F). Furthermore, WB assay and IHC staining of the xenograft tissues obtained from the nude mice confirmed that the knockdown of circ_0009043 can effectively prevent the expression of DNAJB4, while circ_0009043 upregulation enhanced the expression of DNAJB4 expression (Fig. 9G-H). Moreover, TUNEL assay represented that circ_0009043 overexpression (OE-Circ) resulted in much more apoptosis of cells than that in the control (OE-CTRL). However, the NSCLC cell’s apoptosis considerably decreased in the circ_0009043 knockdown group (shCirc), as compared to the shCTRL group (Fig. 9I-J). Western blotting was also carried out for evaluating the changes that occur in Bax, Bcl-2, and Cytochrome C expression. These results are almost consistent with the previously mentioned experiment on the cells (Fig. 9K). The overall results demonstrated that overexpression of circ_0009043 could suppress the growth as well as differentiation of NSCLC. We further confirmed that circRNA 0,009,043 targets the miR-148a-3p / DNAJB4 pathway to suppress the NSCLC *in vivo*.

## Discussion

Although initially disregarded as unimportant byproducts of aberrant splicing events, the advent of high-throughput sequencing and bioinformatics approaches has revealed that circRNAs are widely expressed in a diverse array of cell types and tissues in which they play important functional roles [18]. Differential circRNA expression has been reported in many cancers, and particular circRNA species have been shown to influence cellular invasivity, metastatic progression, proliferation, and differentiation, in addition to being linked to disease status and patient prognostic outcomes [8, 19, 20]. In cervical cancer, for example, circRNA_0000285 and hsa_circ_0000745 have been shown to offer value as prognostic biomarkers [21, 22], while circ_0081001 may offer diagnostic value in osteosarcoma [23]. Here, circ_0009043 was found to be downregulated in NSCLC tissues and cells, thus suggesting it to be an important regulator of NSCLC development.

In prior reports, circ_0009043 was found to be upregulated in grade 3 noncancerous endometrial tissues relative to grade 1–2 noncancerous endometrial tissues [16]. The present study was the first to report on the functional importance of circ_0009043 in NSCLC. Specifically, circ_0009043 downregulation was evident in NSCLC and such downregulation was predictive of poorer NSCLC patient overall survival. Functionally, circ_0009043 knockdown augmented the proliferation of NSCLC cells, and *in vivo* xenograft assays confirmed that the upregulation of circ_0009043 was sufficient to suppress NSCLC tumor growth. Together, these results thus support a role for circ_0009043 as a tumor suppressor circRNA that interferes with NSCLC development.

Despite their lack of coding potential, miRNAs can prevent translation or induce mRNA degradation through sequence-specific binding to the 3’-untranslated regions of target mRNAs [24, 25]. Specific miRNAs have been shown to regulate the pathogenesis of many diseases and to influence normal tissue homeostasis [26, 27]. Functionally, miRNAs play a central role in tumor cell proliferation, metastasis, development, and invasion [28, 29]. Many circRNAs contain multiple miRNA binding sites such that they can serve as de facto miRNA sponges, sequestering these non-coding RNAs and thereby influencing cancer development [8, 10, 30]. In gastric cancer, for example, circ-PVT1 is upregulated and sequesters miR-124-3p, thereby upregulating ZEB1 expression and promoting tumor cell resistance to paclitaxel treatment [31]. Circ-VANGL1 overexpression in bladder cancer can also drive oncogenesis via the miR-605-3p-VANGL1 axis [32]. Notable roles for circRNAs as competing endogenous RNAs have also been reported in breast, ovarian, and colon cancers [33–35]. Potential miRNA binding partners of circ_0009043 were thus identified, with bioinformatics analysis revealing miR-148a-3p binding sites within this circRNA. Luciferase reporter assays then confirmed the ability of circ_0009043 to bind miR-148a-3p. This was noteworthy given that miR-148a-3p has previously been reported to exert tumor suppressor activity in several cancers [36, 37], and differential miR-148a-3p expression has been observed in various malignancies such that it is upregulated in certain cancer types while downregulated in others [38–40]. Functionally, miR-148a-3p has been reported to play a role in shaping cellular development and differentiation, shaping tumor development through the regulation of particular signaling pathways [41]. Previously, miR-148a-3p upregulation has been reported in glioma and osteosarcoma [42, 43], consistent with its importance in oncogenic settings. Here, miR-148a-3p was found to promote NSCLC cell proliferative activity while preventing apoptotic death, functioning at least in part via targeting the DNAJB4 mRNA and thus preventing it from negatively regulating NSCLC progression.

HSPs are frequently identified as promising therapeutic targets and relevant biomarkers in various forms of cancer [44, 45]. DNAJB4 (HLJ1) is an HSP40 family member that has previously been identified as a tumor suppressor gene in lung, colon, and breast cancer patients [46–48], and that has also been specifically identified as a promising therapeutic target and putative biomarker in NSCLC [49]. Consistently, the results of this study revealed that circ_0009043 overexpression resulted in DNAJB4 upregulation, whereas circ_0009043 downregulation had the opposite effect. Notably, the overexpression of miR-148a-3p was sufficient to reverse the impact of circ_0009043 on DNAJB4 upregulation.

## Conclusions

**Fig. 10:**
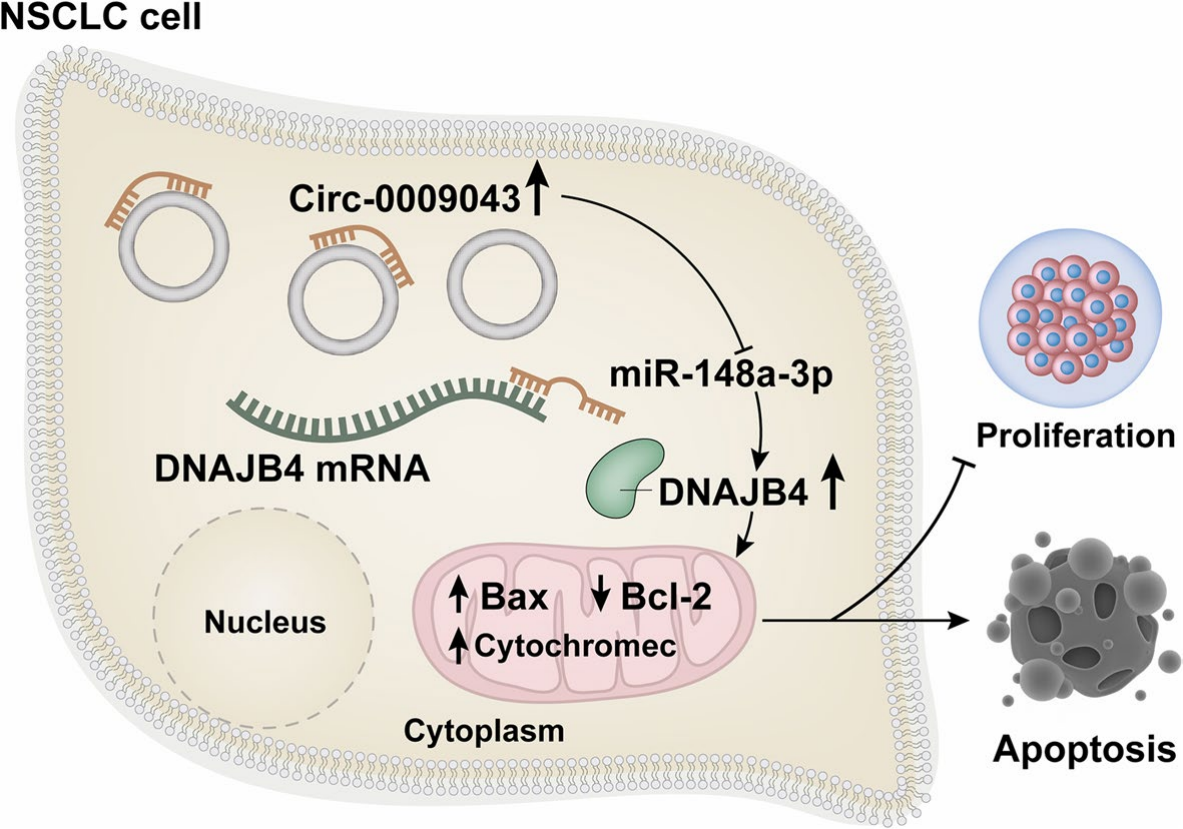
A graphic for the role of circ_0009043 in suppressing the development of non-small-cell lung cancer (NSCLC) via the miR-148a-3p / DNAJB4 pathway. circ_000904 is downregulated in NSCLC cells and that, when expressed, this circRNA can serve as a molecular sponge to sequester miR-148a-3p, thereby leading to DNAJB4 upregulation and the suppression of NSCLC tumor growth

Together, these results revealed that circ_000904 is downregulated in NSCLC cells and that, when expressed, this circRNA can serve as a molecular sponge to sequester miR-148a-3p, thereby leading to DNAJB4 upregulation and the suppression of NSCLC tumor growth (Fig. 10). These results demonstrate the tumor suppressor activity exerted by circ_0009043 in NSCLC cells and suggest that it may offer value as a diagnostic/prognostic biomarker in patients with NSCLC as well as a viable target for therapeutic intervention.

## Data Availability

The data and study materials that support the findings of this study will be available to other researchers from the corresponding authors on reasonable request.

## Abbreviations

circRNAs: Circular RNAs
NSCLC: Non-small-cell lung cancer
HSP: Heat shock protein
DNAJB4: DnaJ heat shock protein family (Hsp40) member B4
RIP: RNA immunoprecipitation
miRNAs: MicroRNAs
FBS: Fetal bovine serum
CCK-8: Cell Counting Kit-8
TUNEL: Terminal deoxynucleotidyl transferase-medimed dUTP nick-endlabeling
qRT-PCR: Quantitative real-time reverse transcription PCR
FITC: Fluorescein isothiocyanate
IHC: Immunohistochemical
